# Macroscopic cerebral energy efficiency corresponds to neuron reorganization in awake and anesthetized mice

**DOI:** 10.3389/fnins.2026.1949911

**Published:** 2026-10-09

**Authors:** Da Wang, Hui Li, Jinggui Gao, Yifan Zeng, Mengyang Xu, Binshi Bo, Mengchao Pei, Zhifeng Liang, Ning Zhou, Garth J. Thompson

**Affiliations:** 1 iHuman Institute, ShanghaiTech University, Shanghai, China; 2 School of Life Science and Technology, ShanghaiTech University, Shanghai, China; 3 Department of Neurology, The First Affiliated Hospital of Shenzhen University, Shenzhen Second People's Hospital, Shenzhen, China; 4 Center for Excellence in Molecular Cell Science, Shanghai Institute of Biochemistry and Cell Biology, Chinese Academy of Sciences, Shanghai, China; 5 University of Chinese Academy of Sciences, Beijing, China; 6 CAS Center for Excellence in Brain Science and Intelligence Technology, Institute of Neuroscience, Chinese Academy of Sciences, Shanghai, China; 7 School of Pharmacy, Anhui University of Chinese Medicine, Hefei, China

**Keywords:** calibrated fMRI, cerebral energy efficiency, CMRO2, relative cost, relative power

## Abstract

**Introduction:**

Non-invasive imaging of brain function and energy supply is crucial for diagnosing and treating brain disorders. Conventional imaging struggles to capture altered relationships between energy supply and demand caused by brain diseases. A novel method, thus far only tested non-invasively in human subjects, is to calculate relative power (rPWR) and relative cost (rCST) to assess cerebral energy efficiency. However, whether rPWR/rCST tracks individual changes and relate to neural activity remains unproven.

**Methods:**

Our study examines awake and anesthetized male mice and compares rPWR/rCST with invasive two-photon microscopy.

**Results:**

We found that rPWR/rCST distributions were similar between awake mice and humans, but changed in anesthetized mice, indicating a shift in the brain’s economic balance. Furthermore, changes in rPWR/rCST were linked to the reorganization of microscopic neural networks, observed with two-photon microscopy.

**Discussion:**

Our work highlights the potential of rPWR/rCST for medical applications, and that neural network reorganization is linked to the brain’s economic balance.

## 1 Introduction

The brain is highly energy-demanding, consuming about 20% of the body’s oxygen despite comprising only 2% of its weight (Clarke, 1999; Attwell and Laughlin, 2001; Magistretti and Allaman, 2015). Altered energy metabolism serves as a biomarker for neurodegenerative disease diagnosis/treatment (Picca et al., 2021; Minoshima et al., 2022). A strong relationship exists between energy utilization and neuronal activity (Lundgaard et al., 2015; Bonvento and Bolanos, 2021).

Brain development, both evolutionary and individual, balances minimizing wiring costs with optimizing neuronal connectivity (Rocher et al., 2003; Liang et al., 2022). Wiring costs, including material and energy expenditures, are minimized through topological optimizations such as shortest-path neuronal connections (Karbowski, 2007) and small-world network structures that enhance efficiency (Henderson and Robinson, 2011; Samu et al., 2014). These structures ensure rapid, accurate information transmission, optimizing cognitive function (He et al., 2009; Bullmore and Sporns, 2012). Neurodegenerative diseases, such as Alzheimer’s, disrupt these networks, impairing cognitive efficiency (Stam et al., 2007). Although advanced-stage metabolic disorders are relatively obvious (Lajoie et al., 2017), early metabolic alterations remain subtle, limiting clinical diagnosis due to current low detection sensitivity (Smailagic et al., 2015). Measuring brain energy economics offers promise for early detection when intervention can still improve outcomes (Sabbagh et al., 2020; Porsteinsson et al., 2021).

Functional magnetic resonance imaging (fMRI) is a widely used non-invasive method for investigating brain energy dynamics, utilizing the blood oxygen level-dependent (BOLD) signal to infer neural activity. The BOLD signal is a combination of the cerebral metabolic rate of oxygen (CMR_O2_), cerebral blood flow (CBF), and cerebral blood volume (CBV) (Biswal et al., 1995). “Calibrated fMRI” techniques can measure CMR_O2_ by combining BOLD and either CBF or CBV measurements during gas (typically CO_2_) administration in a metabolic model (Bulte et al., 2012; Gauthier and Hoge, 2013). Instead of gas calibration, “relaxometry-based calibrated fMRI” uses magnetic relaxation maps, thus allowing new animal experiments and clinical studies which were previously not possible (Xu et al., 2022; Zhang et al., 2022).

Microscopic alterations significantly influence macroscopic brain metabolism (Ardestani et al., 2016). In Alzheimer’s disease models, neuronal injury in affected cortical regions leads to astrocyte damage (Verkhratsky et al., 2017; Toledano-Diaz et al., 2022), contributing to vascular dysfunction and reduced CBF (Amtul et al., 2020), ultimately impacting CMR_O2_ (Yang et al., 2023). Understanding the interplay between metabolic activity and brain function can help elucidate neurodegenerative mechanisms.

Many studies link brain activity with energy consumption, yet neurodegenerative diseases disrupt this relationship (Mosconi, 2005; Magistretti, 2006). Precise measurements may reveal neurodegenerative mechanisms, improving early diagnosis. Relative power (rPWR) and relative cost (rCST) were developed to compare energy supply and demand, where rPWR reflects total neurometabolic activity and rCST quantifies energy efficiency (Shokri-Kojori et al., 2019). These metrics derive from fMRI-based local functional connectivity density (lFCD) as a proxy for energy demand and metabolic markers such as the cerebral metabolic rate of glucose (CMR_glc_) from ^18^F-fluorodeoxyglucose positron emission tomography (FDG-PET) or CBF from MRI. While this methodology has proven sensitive to alcohol-induced metabolic changes (Shokri-Kojori et al., 2019), key questions remain. First, it is unclear whether rPWR and rCST track neurometabolic changes within individuals across states. Second, the precise neural activity underlying these measurements remains unknown, limiting their application to therapies targeting neuronal function. Third, rPWR and rCST have not been calculated in an animal model and thus, fourth, it is unknown whether they can translate between animal models and human subjects.

To address these gaps, we used an awake versus anesthetized mouse model, integrating non-invasive fMRI with invasive two-photon calcium imaging microscopy (2PCI). To our knowledge, this is the first study to apply rPWR/rCST analysis in a mouse model and also the first study to combine it with two-photon calcium imaging, thereby linking macroscopic cerebral energy efficiency with microscopic neuronal network organization. We hypothesized that anesthesia would induce significant alterations in energy utilization efficiency and neuronal network organization. Our findings offer valuable insights into neurometabolic regulation.

## 2 Materials and methods

This study incorporates two distinct types of data, MRI data and two-photon data. The MRI data used in this study were combined from two previously published works (Xu et al., 2022; Wang et al., 2024). The primary data analysis methods and results of MRI in this study, including rPWR, rCST, and brain region selection, are unique to this study and have not been published elsewhere. The two-photon calcium imaging data were newly acquired in an independent mouse cohort as an MRI-guided follow-up experiment, focusing on regions selected from the fMRI rPWR/rCST analysis.

### 2.1 MRI experiments

#### 2.1.1 Animals

The experimental animals were sourced from the Shanghai Experimental Center. Fourteen male adult C57BL/6 mice, aged 7 to 9 weeks and weighing between 20 and 30 grams, were used. All mice were housed in strictly controlled environments, maintaining a humidity level of 50 ± 10% and a temperature range of 20 to 26 °C. Mice were exposed to a regular 12-h light/12-h dark cycle and had unrestricted access to rodent chow and water. All animal experiments were approved by the Institutional Animal Care and Use Committee of ShanghaiTech University and the Institute of Neuroscience, Chinese Academy of Sciences, China, in accordance with the governmental regulations of China for the care and use of animals.

The preparation of the animals followed the protocol as described by Xu et al. (2022). After being anesthetized with isoflurane, mice underwent head surgery to expose the skull and affix a head-fixing device. After that, the mice were secured in a head holder and trained to become accustomed to the MRI imaging noise and environment. One week after collecting awake imaging data (Awake), the mice were anesthetized with dexmedetomidine (Dex) to capture images in the anesthesia state. For the Dex session, mice were induced with isoflurane, followed by intraperitoneal dexmedetomidine bolus injection at 0.025 mg/kg. Isoflurane was discontinued 5 min after dexmedetomidine injection, and dexmedetomidine was continuously infused subcutaneously at 0.05 mg/kg/h starting 10 min after injection. Body temperature was monitored with a rectal probe and maintained at 37.0 ± 0.5 °C using a warm-water pad. Data from these two sessions (Awake and Dex) for a total of 14 mice were used.

#### 2.1.2 MRI imaging

All animals were scanned on a 9.4 T MRI system, using a 4-channel cryogenic mouse head coil for receive and an 86-mm volume coil for transmit (Bruker Biospin) (Supplementary Figure S1a). Localization scans were done first to center the mouse brain within the imaging field. Different sequences were then used to acquire images, and the full sequence names and details are provided in Table 1. Parameters imaged included the brain structure (T2 Turbo RARE), maps for the T_2_ and T_2_* magnetic relaxation constants (MSME and MGE, respectively), CBF signals (pCASL), and fMRI BOLD signals (EPI) from the mouse brain.

**Table 1:** MRI sequences and their parameters.

Sequence	Echo time (TE)	Repetition time (TR)	Field of view (FOV)	Image size	Number of slices	Slice thickness	Other non-default parameters
T2 Turbo RARE	334 ms	3,300 ms	16 × 16 mm^2^	256 × 256	20	0.5 mm	Averages:2
MGE	2.15–17.23 ms	500 ms	22 × 16 mm^2^	110 × 80	20	0.5 mm	Echoes: 8, averages: 4
MSME	10-100 ms	3,000 ms	22 × 16 mm^2^	110 × 80	20	0.5 mm	Echoes: 10, average: 1
pCASL	22 ms	4,000 ms	71 × 71 mm^2^	15.975 × 15.975	20	0.5 mm	Label duration: 3000 ms Post labeling delay: 600 ms Pairs of label/control: 80 Inversion efficiency: 0.59
EPI	15 ms	1,000 ms	15.975 × 12.15 mm^2^	71 × 54	20	0.5 mm	Repetitions: 600

RARE, rapid acquisition with relaxation enhancement; MGE, multiple gradient echo; MSME, multi slices multi echo; pCASL, pseudocontinuous arterial spin labeling.

#### 2.1.3 fMRI processing method

As detailed in Wang, et al. fMRI BOLD data underwent preprocessing and analysis using custom MATLAB scripts, including motion correction, slice timing adjustment, anatomical image registration to the mouse brain template (brain regions of interest and abbreviations are shown in Supplementary Table S1), spatial smoothing, and bandpass filtering of voxel time series between 0.01 and 0.3 Hz (Wang et al., 2024).

For noise removal, epochs with low signal-to-noise ratios for brain networks were excluded, and group independent component analysis was used to remove noise components and enhance the quality of the BOLD signal. As the primary somatosensory cortex of the forelimb (S1FL) exhibits a strong correlation between the left and right hemispheres, lacking correlation in S1FL is an indicator of disrupted brain networks in general, potentially signifying an underlying physiological issue. Thus, data with Pearson’s correlation coefficient (PCC) less than 0.3 between the bilateral S1FL were excluded as several previous studies have done (Johnson et al., 2018; de Arce et al., 2023; Li et al., 2024) To reduce noise and improve the quality of the BOLD signal, group independent component analysis (ICA) was conducted using GIFT toolbox^1^ as several previous studies have done (Han et al., 2019; Wang et al., 2024). The signal from ventricles and white matter, components 1, 2,4, and 8 were removed (Supplementary Figure S2).

#### 2.1.4 Local FCD estimation

Functional connectivity density mapping (FCD) mapping is a voxel-wise graph theoretical approach that aims to identify hubs within the brain’s networks. FCD involves counting the number of local or global functional connections for each voxel in the brain, utilizing Pearson’s correlation coefficient as a measure of connectivity (Tomasi and Volkow, 2010). Following Shokri-Kojori et al., here local FCD (lFCD) was used to calculate the number of voxels linked with nearby voxels (Cohen et al., 2017). A key distinction in local functional connectivity density (lFCD) calculation between the present study and Shokri-Kojori et al.’s work lies in two species-specific parameters: Shokri-Kojori et al. utilized a Pearson correlation coefficient (PCC) threshold of 0.6 and a 5 mm-diameter spatial neighborhood for human brain analyses, whereas our mouse-centric study adopted a PCC threshold of 0.25 and a 2 mm-diameter spatial neighborhood—adjustments tailored to the mouse brain’s anatomical scale (Cohen et al., 2017; Shokri-Kojori et al., 2019) based on our previous work (Wang et al., 2024).

#### 2.1.5 Removal of signals related to temporal signal to noise variation

As per the methods of Shokri-Kojori et al., after preprocessing and removing the signal from ventricles and white matter as described above, the voxel-wise temporal signal to noise (tSNR) of fMRI data were calculated using the mean to standard deviation ratio of the raw fMRI time series (Shokri-Kojori et al., 2019). Here, rPWR and rCST were calculated by FCD and CMR_O2_, to improve the quality of rPWR and rCST, we calculated the correlation coefficient between tSNR and lFCD, CMR_O2_, rPWR, and rCST. The brain regions that had a significant correlation with rPWR and rCST were removed for further analysis and were shown in black on the brain maps in Figure 1.

**Figure 1:**
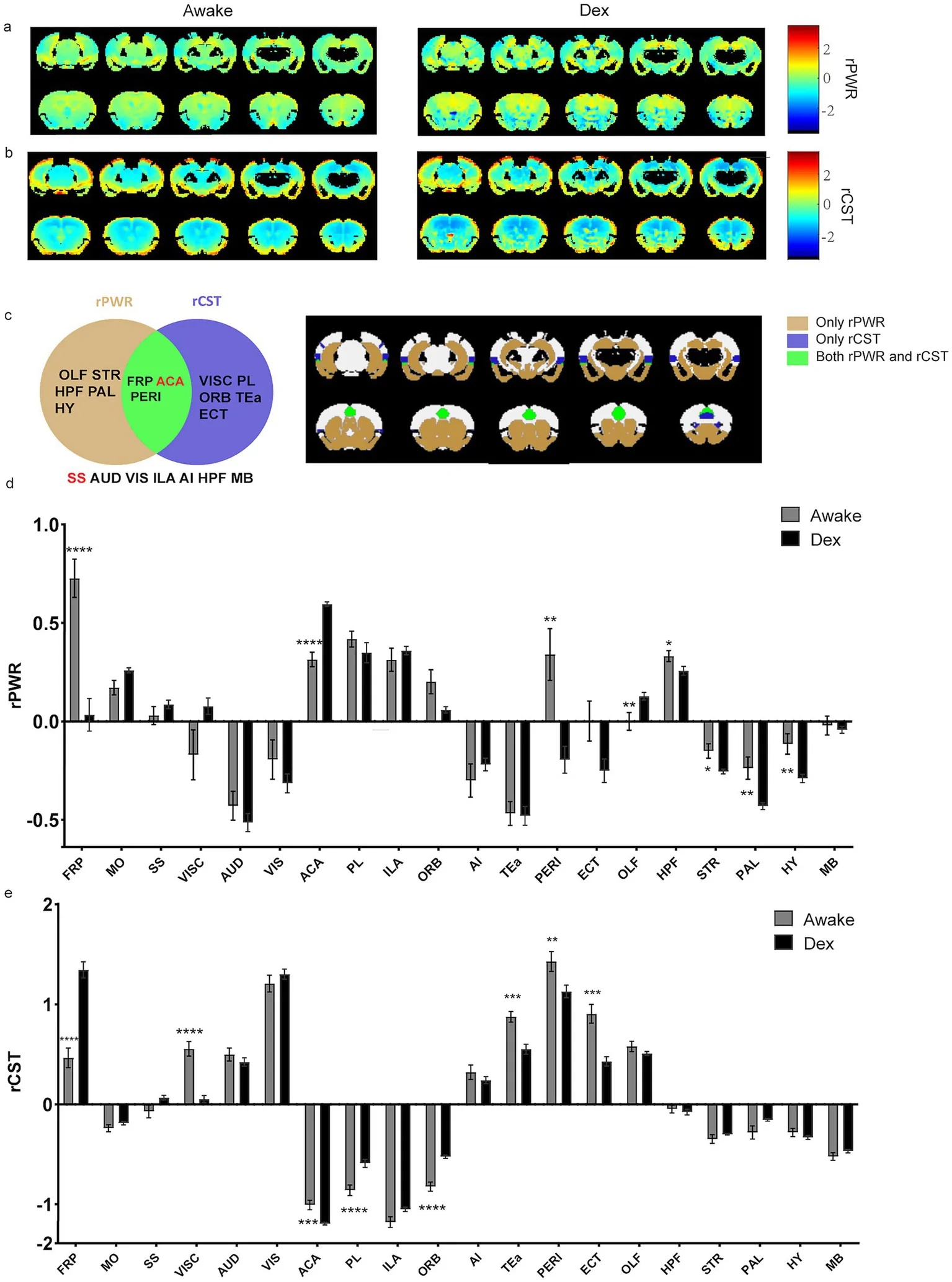
rPWR and rCST under Awake and Dex conditions. **(a,b)** Average rPWR **(a)** and rCST **(b)** across all mice in awake and Dex groups in 10 coronal slices, from caudal to rostral (*n* = 14 mice). Brain regions where CMR_O2_ or lFCD significantly correlated with tSNR (see Materials and methods and Supplementary Table S5) were removed and are depicted as black. **(c)** Summary of rPWR and rCST statistical results. The brown and blue circles indicate brain regions that exhibit statistically significant differences between Awake and Dex in rPWR and rCST, respectively. The intersection of these two circles indicates areas that exhibit statistically significant differences in both rPWR and rCST, and regions below the circles exhibited no statistically significant differences in either rPWR or rCST. **(d,e)** Comparison of rPWR **(d)** and rCST **(e)** averaged for 20 brain regions. Data shows mean ± s.e.m. (paired-t test, * indicates *p* < 0.05, ** indicates *p* < 0.01, *** indicates *p* < 0.001, **** indicates *p* < 0.0001; specific information on t-values and *p*-values is provided in Supplementary Table S5). FRP = frontal pole; MO = somatomotor areas; SS = somatosensory areas; VISC = visceral areas; AUD = auditory areas; VIS = visual areas; ACA = anterior cingulate area; PL = prelimbic area; ILA = infralimbic area; ORB = orbital area; AI = agranular insular area; TEa = temporal association areas; PERI = perirhinal area; ECT = ectorhinal area; OLF = olfactory area; HPF = hippocampus formation; STR = striatum; PAL = pallidum; HY = hypothalamus; MB = midbrain.

#### 2.1.6 Calibrated fMRI

The relaxometry-based calibrated fMRI method was used to obtain relative CMR_O2_, shown in Equation 1. This method was used as it does not require a gas challenge (Xu et al., 2022). As per Xu et al., p813, “R_2_′ is the (positive) increase in magnetic relaxation rate (Xu et al., 2022), *A* is a field-dependent magnetic susceptibility constant (Boxerman et al., 1995). β is the exponent in the power-law dependence between BOLD signal and the deoxyhemoglobin concentration in blood ([dHb]) which depends on blood vessel size (Davis et al., 1998).” Grubb’s constant α is a proportionality exponent describing the power-law relationship between CBF and blood volume (CBV), quantifying how CBF scales with CBV changes (Grubb et al., 1974). The value of *A* was determined by referencing the awake state CMR_O2_ reported previously of 116 μmol/100 g/min (Cao et al., 2017). Using the average of R_2_’ and CBF measurements from awake mice and Equation 1, we calculated *A* = 69.3257. Note that this *A* value was only used as an estimate to allow per-subject calculation of rCMR_O2_, we do not claim that values produced using this *A* value are quantitative CMR_O2_. In accordance with previous work in mice, we used *α* and *ß* of 0.2 and 0.8, respectively (The *ß* used was the average of the *ß* brain map) (Xu et al., 2022).


$$\mathrm{CM}R_{O2}=4*{\left(\frac{R_{2}^{\prime }}{A}\right)}^{\frac{1}{\beta }}*{\left(\mathrm{CBF}\right)}^{1-\frac{\alpha }{\beta }}$$ (1)

#### 2.1.7 Calculation of rPWR and rCST

rPWR and rCST were estimated following Shokri-Kojori et al.: voxel-wise rPWR and rCST are derived from a 45° counterclockwise rotation of the axes of standardized log(lFCD) and (CMR_O2_) (Equation 2)^2^ (Shokri-Kojori et al., 2019). We used CMR_O2_ as the metabolic supply and lFCD as the metabolic demand. Shokri-Kojori obtained similar rPWR and rCST results whether they used CMR_glc_ or CBF, therefore we expect CMR_O2_ may produce similar results as it is a gross metabolic measurement like CMR_glc_, reflects the supply of fresh blood like CBF, and may better reflect the metabolic supply for the BOLD signal than either of these (Hoge et al., 1999). As described above, the CMR_O2_ of certain brain regions correlated with temporal signal to noise and were removed prior to rPWR and rCST calculation. Voxelwise analysis was conducted within the Allen brain mask, to focus on functionally relevant regions.


$$\left[\;\begin{array}{c} \text{rPWR}\\ \text{rCST} \end{array}]=[\begin{array}{cc} \cos\frac{\pi }{4} & \sin\frac{\pi }{4}\\ -\sin\frac{\pi }{4} & \cos\frac{\pi }{4} \end{array}][\begin{array}{c} z(\log(\text{lFCD}))\\ z(\mathrm{CM}R_{O2}) \end{array}\right]$$ (2)

### 2.2 Two-photon calcium imaging experiments

#### 2.2.1 Animals

Two-photon imaging was conducted only after results were known for rPWR and rCST. We selected one brain region where rPWR and rCST significantly changed between Awake and Dex groups and one region where changes were not significant. In choosing these regions, we also considered the degree of change, looking for areas with large changes (i.e., significant) and areas with small changes (i.e., non-significant) as well as the feasibility of imaging these regions with two-photon imaging. For Dex two-photon imaging, the same dexmedetomidine anesthesia protocol as described for the MRI Dex session was used. A parameter comparison between MRI and two-photon imaging experiments is shown in Supplementary Table S2.

Four male C57BL/6 J mice (2 mice per each chosen brain region), aged 7 to 9 weeks and weighing between 20 and 30 grams were purchased from the Animal Core Facility of the corresponding authors’ institution. This number was chosen based on existing two-photon imaging studies (Kondo et al., 2017). All mice were housed in strictly controlled environments, maintaining a humidity level of 50 ± 10% and a temperature range of 20 to 26 °C. Mice were exposed to a regular 12-h light/12-h dark cycle and had unrestricted access to rodent chow and water. All animal experiments were performed in accordance with procedures approved by the Institutional Animal Care and Use Committee of the corresponding authors’ institution and ﻿were consistent with local governmental regulations for the care and use of animals. Prior to commencing the experiments, all mice were quarantined for a week to ensure they were free of specific pathogens.

#### 2.2.2 Two-photon calcium imaging

For the two-photon calcium imaging (2PCI), a precision micro handheld skull drill was employed to fashion a 4 mm-diameter cranial window precisely positioned above the virus injection site or its symmetrical counterpart relative to the sagittal suture. Following this, a glass slide measuring 4 mm in diameter and 100 μm in thickness, along with a drug administration cannula, were gently inserted into the cranial window and securely adhered using biological tissue glue. Mice were allowed to recover for at least one week after cranial window, coverslip, and cannula implantation, with the recovery period extended when necessary based on postoperative condition and imaging-window quality. Then an imaging baseplate was meticulously placed on the cranial window and firmly adhered with dental acrylic resin. The miniature two-photon microscope (FHIRM-TPM V2.0, Transcend Vivoscope Biotech), although detachable, features a bracket that is permanently affixed to the baseplate situated above the cranial window. Prior to imaging, both the bracket cover and the protective gel covering the cranial window were carefully removed. Subsequently, the headset was seamlessly mounted onto the bracket and securely locked in place using an M2 screw, ensuring stability and precision during the imaging process.

100 nL of AV2/9-hsyn-GCaMP6f-WPRE-pA virus was injected at a rate of 50 nL/min. After recovery from virus injection, mice were habituated to awake head-fixed 2PCI experiments. The experimental plan is shown in Supplementary Figure S3a. The injection sites selected (see Results section for the selection process) are shown in Supplementary Figures S3b,c. The emission filter of GCaMP6f has a wavelength range of 500 to 550 nm and an excitation wavelength of 920 nm. Image data were acquired using Imaging software (GINKGO-MTPM, Transcend Vivoscope Biotech) at a frame rate of 9.76 Hz, total imaging time was 10 min. Two full sessions were recorded for each mouse and each condition. The Field of view (FOV) size was 420 × 420 μm (512 × 512 pixels). The average power delivered to the brain was less than 110 mW. Before and after each recording, the focal plane and imaging position were checked and manually re-aligned to the initial reference image when necessary, to minimize out-of-focus effects during awake imaging. Upon acquiring the calcium fluorescence data, immunofluorescence staining was subsequently performed to confirm the presence of injection of virus in ACA (anterior cingulate area, Supplementary Figure S3b), and in the SS (somatosensory areas, Supplementary Figure S3c).

#### 2.2.3 2PCI processing

Somatic regions were identified by STNeuroNet^3^, and manual calibration in imageJ^4^.

Calcium imaging data were analyzed with custom-written MATLAB software. The fluorescence signal of each neuron was extracted from identified neurons using the annular ring subtraction (ARS) algorithm (Zong et al., 2021), and the fluorescence change relative to the baseline (“∆F/F”) was calculated as the calcium transients. Motion artifacts in awake mouse imaging were corrected using the NoRMCorre algorithm, a piecewise rigid motion correction method (Pnevmatikakis and Giovannucci, 2017). The fluorescence baseline (F0) for ∆F/F calculations was defined using a 15th percentile approach, where pixel values within each region of interest (F_ROI_) were sorted in ascending order, and the 15th percentile value was selected to minimize the impact of transient fluctuations and extreme pixel biases (Liang et al., 2018). Neurons with poor-quality signals and fluorescence traces were excluded based on a signal to-noise ratio (SNR) threshold, removing neurons with SNR < 3 in their ∆F/F curves. Calcium events were defined using MATLAB’s “findpeaks” function, supplemented by manual visual inspection. The frequency of calcium events was quantified as the number of occurrences per cell per minute. The amplitude of calcium transients was measured by the change in fluorescence (ΔF/F), and the duration was characterized by the full width at half maximum (FWHM) amplitude. All of these values were calculated using custom-written MATLAB scripts.

#### 2.2.4 Small-worldness calculation

For each mouse, three representative regions of interest (ROIs) were randomly selected across the imaged brain area. Each ROI contained 8–20 neurons. 24 resulting ROIs (two brain regions, two mice per brain region, two sessions per mouse, three ROIs per session) had small-worldness calculated therein.

Small-worldness (SW) was characterized by high global cluster coefficient *CC* and short average path length *L* (Equation 3) (Scharwachter et al., 2022). Cluster coefficient measured the degree to which nodes in the network tend to cluster together (Equation 4) (Scharwachter et al., 2022). For a node i , the local clustering coefficient $\;CC_{i}$ is the ratio of the number of edges $E_{i}$ between the node’s neighbors to the maximum possible number of edges between them. *k_i_* is the number of connections between node i and other nodes. CC is global cluster coefficient, is the average of $CC_{i}$ . The average path length (L) is the mean of shortest path lengths between all pairs of nodes in the network (Equation 5). *N* is the number of nodes. $\;n_{\mathrm{hj}}(i)$ is the shortest number of paths between node j and node h through node i , $n_{\mathrm{hj}}\;$ is the shortest number of paths between node j and node h .


$$\begin{array}{c} \mathrm{SW}=\frac{\mathrm{CC}}{L} \end{array}$$ (3)

$$\begin{array}{c} CC_{i}=\frac{2E_{i}}{k_{i}(k_{i}-1)} \end{array}$$ (4)

$$\begin{array}{c} L=\frac{1}{N(N-1)}\Sigma_{n\neq j\neq i}\frac{n_{\mathrm{hj}}(i)}{n_{\mathrm{hj}}} \end{array}$$ (5)

### 2.3 Statistical analysis

Paired *t* tests were performed, examining the effect of anesthesia on metrics derived from MRI and 2PCI data. The metrics upon which significance testing was conducted were rPWR, rCST, lFCD and CMR_O2_ from MRI data, and frequency, amplitude, duration, and small-worldness from 2PCI data. The *p* values were corrected based on the sequential goodness of fit metatest for multiple comparisons (Carvajal-Rodríguez et al., 2009).

To assess the robustness of the rPWR and rCST results to parameter selection, two sensitivity analyses were performed. First, the PCC threshold used for lFCD estimation was varied from the primary value of 0.25 to 0.22 and 0.28, and the neighborhood size was varied from the primary value of 2.0 mm to 1.9 and 2.1 mm. All nine combinations of these values were evaluated. Second, the CMRO2 model parameters A, *β*, and α were independently varied by ±10% around their primary values of 69.3257, 0.80, and 0.20, respectively, yielding 27 parameter combinations. For every parameter combination, lFCD or CMR_O2_, as applicable, and the downstream rPWR and rCST measures were recalculated. Robustness was assessed using Pearson correlations between the Awake-Dex difference maps obtained with each alternative setting and those obtained with the primary setting. Regional paired t-test *p* values for ACA and SS are reported descriptively in Supplementary Tables S3,S4; formal statistical inference was based on the primary analysis with SGoF correction for multiple comparisons.

## 3 Results

### 3.1 Relative power (rPWR) and relative cost (rCST) derived from functional connectivity density (lFCD) and cerebral metabolic rate of oxygen (CMR_O2_)

Herein we calculate rPWR and rCST using CMR_O2_ as the “metabolic supply” and lFCD as the “metabolic demand.” Previous work also used lFCD for demand, but used either CMR_glc_ or CBF for supply (Shokri-Kojori et al., 2019). CMR_O2_ in healthy subjects is highly correlated to both CMRglc and CBF (Lying-Tunell et al., 1980; Zhang et al., 2022) and hypothetically reflects the metabolic supply for BOLD (Hoge, 2012), thus is well-suited as a measurement for local energy supply in the brain (Supplementary Figure S1). In addition, using either CBF or CMR_O2_ also allows measurement of rPWR and rCST using only MRI (Supplementary Figure S1).

We build on our previous work with mouse models comparing the awake state with anesthetized conditions, a state change which is highly useful for elucidating the intricate relationship between energy supply and metabolism (Mortensen et al., 2018; Xu et al., 2022; Wang et al., 2024). Numerous prior studies have demonstrated that anesthesia significantly modulates the tricarboxylic acid (TCA) cycle (Marjańska et al., 2018; Hyppönen et al., 2021), subsequently inducing alterations in CMR_O2_ and CBF (Slupe and Kirsch, 2018). Leveraging the mouse as our primary research subject, we were later able to further probe the neuronal-level changes induced by awake versus anesthetic states, utilizing invasive optical imaging technology.

### 3.2 rPWR and rCST changes between the awake and Dex groups within the same mice

We recorded fMRI, to measure the energy demand using lFCD, and calibrated fMRI, to measure the energy supply using CMR_O2_, from which we calculated rPWR and rCST in two states, awake mice (Awake) and the same mice anesthetized with dexmedetomidine (Dex). A map of the whole brain and a bar plot of average results in 20 brain regions is shown for rPWR and rCST (Figures 1a,b). Our results showed that rPWR and rCST had significant, region-specific state changes between the Awake and Dex states within the same mice (Figures 1c,e). The trends of changes in rPWR and rCST due to anesthesia effects exhibited a distinct pattern. For example, In the anterior cingulate area (ACA) brain region, there was a significant increase in rPWR and a concurrent significant decrease in rCST (*p* = 1.69 × 10^−5^ *t (13)* = 6.61 rPWR, *p* = 8.57 ×10^−4^ *t(13)* = −4.3 rCST). lFCD trended downward in most brain regions, with significant differences restricted to three specific areas (perirhinal area (PERI), ectorhinal area (ECT), hypothalamus (HY)). CMR_O₂_ also showed significant decreases across most regions. (Supplementary Figure S4). In contrast to lFCD and CMR_O2_, the change we observed in rPWR and rCST was very heterogenous, and highlighted specific regions, described below (Figures 1d,e). Specific information on *t*-values and *p*-values is provided in Supplementary Table S5.

### 3.3 Comparison of rPWR and rCST between humans and awake mice

While Shokri-Kojori, et al. focused on human populations (Shokri-Kojori et al., 2019), our study marks the first application of rPWR and rCST in a mouse model. Healthy human data showed higher rPWR in the default mode network (DMN), which indexes high activity and energy demand. This is consistent with the DMN network being the most active and energy demanding network (Buckner et al., 2008; Vaishnavi et al., 2010; Raichle, 2015). Similar to human data, our awake mouse data showed higher rPWR in the ACA region (Table 2), which, in rodents, is believed to have homology with the human DMN (Sforazzini et al., 2014). The highest rPWR between the 20 brain regions was the frontal pole (FRP) region, which can be explained by a high concentration of proteins related to the tricarboxylic acid cycle in the FRP region (Shaw et al., 2020). Lower rCST was also found in the ACA for both humans and mice (Table 2).

**Table 2:** Comparison of rPWR and rCST between awake humans and awake mice.

Observation	Human	Mouse
Higher rPWR	DMN ~ 0.7 (estimated from Figure 2d in Shokri-Kojori et al., 2019). The DMN is active and ﻿metabolically demanding.	ACA = 0.31 (mean): Part of the rodent DMN. FRP = 0.73 (mean): a high distribution of﻿ tricarboxylic acid cycle related proteins.
Lower rCST	ACA < 0	ACA = −1.01

rPWR, relative power; rCST, relative cost; DMN, default mode network; FRP, Frontal pole, cerebral cortex; ACA, anterior cingulate area.

### 3.4 Enhanced differentiation of ACA and SS regions through rPWR and rCST metrics

rPWR and rCST demonstrate a more pronounced divergence in per-brain region changes compared to the lFCD and CMR_O2_. When employing FCD or CMR_O2_, the anterior cingulate area (ACA) and the somatosensory (SS) regions exhibit similar patterns, lacking any significant deviation from the overall dataset (Figs. 2A, B) (Xu et al., 2022; Wang et al., 2024). However, upon the application of rPWR and rCST, a stark contrast emerges between the ACA and SS regions (Figures 2c,d). The ACA, in particular, emerges as an outlier with statistically significant differences, highlighting its distinct response to anesthesia. This observation demonstrates that unique insights are provided by rPWR and rCST, metrics that have not been previously utilized to assess state changes in brain activity. Our findings suggest that these relative metrics offer a more nuanced understanding of brain region dynamics, potentially unveiling distinct neurophysiological signatures that were not apparent with the original metrics.

**Figure 2:**
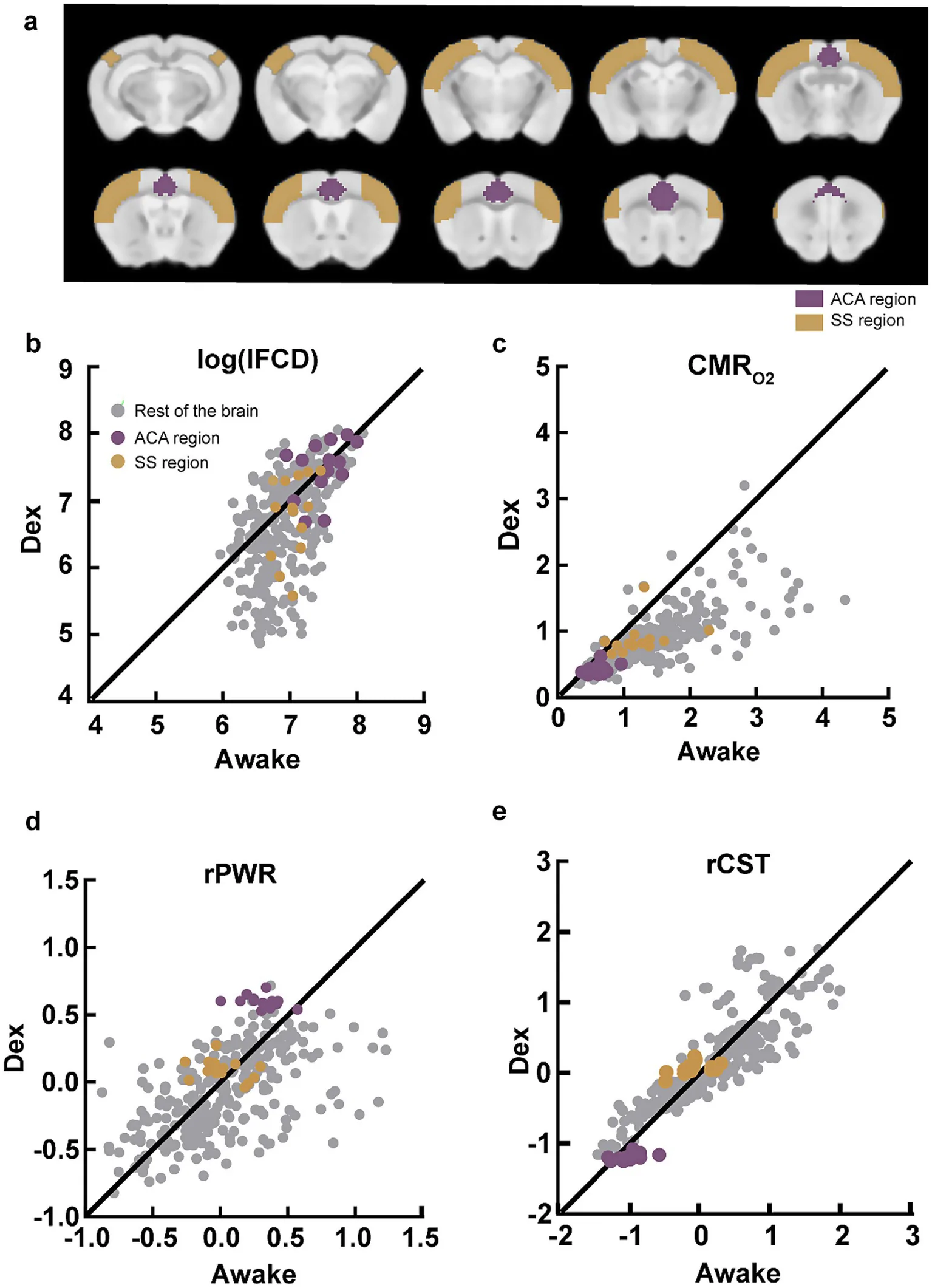
Scatter plots of natural logarithm of lFCD, CMR_O2_, rPWR and rCST. **(a)** Brain map (as per Figures 1a–c) showing the locations of ACA region and SS regions. **(b–e)** Each dot is one mouse’s value in one brain region (*n* = 14 mice). Purple, yellow and gray circles are values from the ACA, SS, and remaining brain regions. Above the diagonal indicates a higher value under anesthesia, and below indicates a higher value when awake. The values on the diagonal are where awake and anesthesia states are approximately equal. lFCD = local functional connectivity density; CMR_O2_ = cerebral metabolic rate of oxygen; rPWR = relative power; rCST = relative cost; ACA = anterior cingulate area; SS = somatosensory areas.

### 3.5 Anesthesia induced a decrease of neuronal activity within the SS and ACA regions

Based on our rPWR and rCST results, we selected the SS region as a region which showed no or minimal response to anesthesia, and the ACA as a region which showed a strong and statistically significant response to anesthesia. We performed 2PCI experiments under Awake and Dex conditions in mice with the imaging field of view located in the SS region and, in a separate cohort of mice, in the ACA region.

Data from 2PCI experiments were analyzed to examine changes in the neuronal activity caused by anesthesia (Figures 3a,b). We measured the frequency, amplitude and duration of calcium transients (Figures 3c–h). We found that within the SS brain region, the frequency and amplitude of calcium transients was significantly decreased under Dex (*p* = 0.011, *t(3)* = −5.61 for frequency, *p* = 0.024, *t(3)* = −4.23 for amplitude). The duration of calcium transients did non-significantly change under Dex. Within the ACA brain region, there was no significant difference for either amplitude or duration between Awake and Dex (though the trend was similar to the SS region), and the frequency was significantly decreased under Dex (*p* = 0.016, *t(3)* = −4.94).

**Figure 3:**
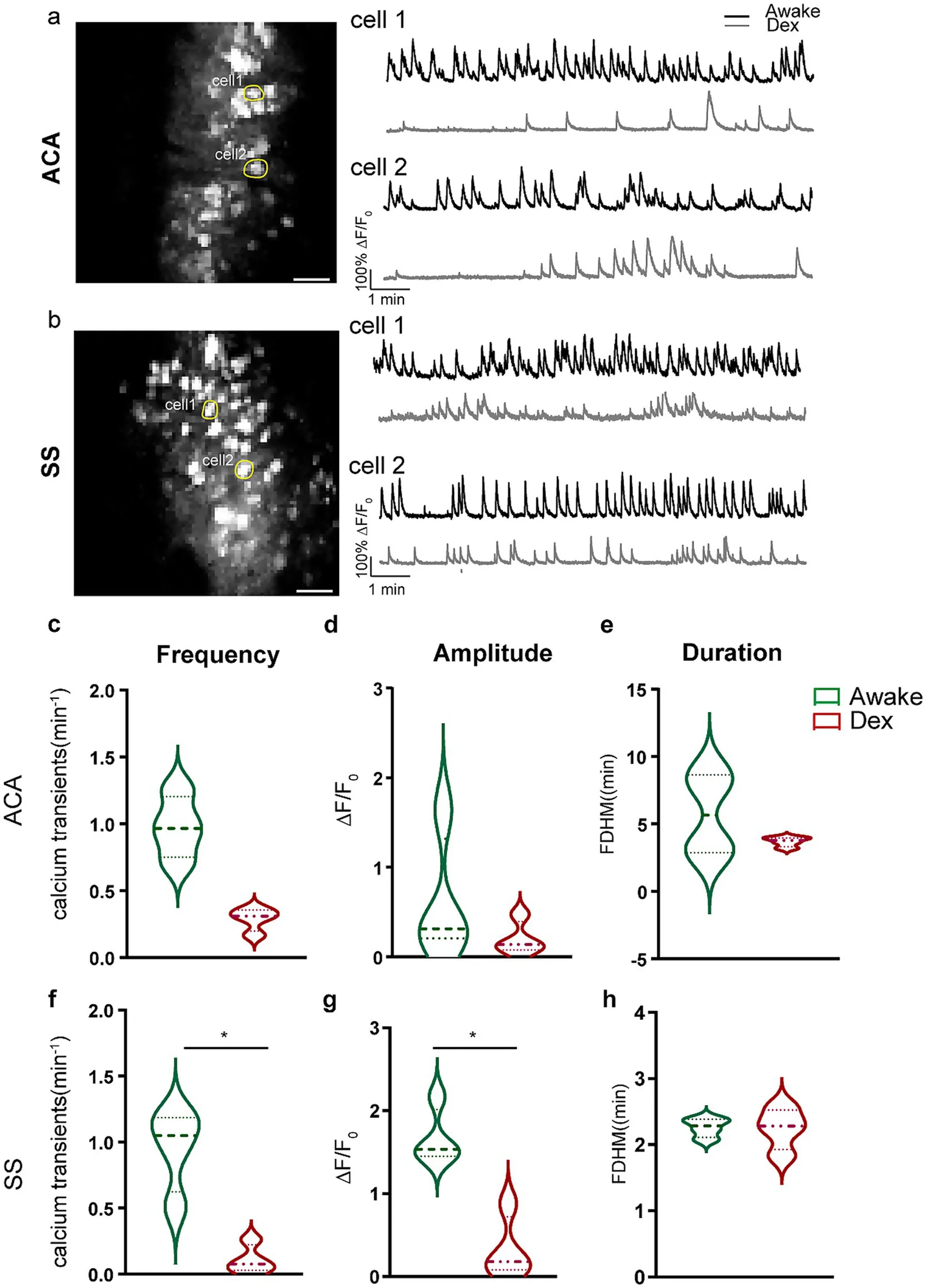
Calcium transients within ACA and SS regions under Awake and Dex conditions. **(a,b)** Single cell calcium intensity within the ACA **(a)** and SS **(b)** brain regions under different anesthesia states. **(c–h)** Frequency, amplitude, and duration of calcium events within the ACA and SS brain regions under the different states. The upper and lower dotted lines represent the third and first quartiles, respectively, and the thick dotted line is the median. Asterisks (*) indicate statistically significant differences (*p* < 0.05) between the awake and anesthetized states in the corresponding brain regions for the corresponding metrics. (paired-*t* test, *p* = 0.0112, *t (3)* = 5.609 in *f*, *p* = 0.0241, *t (3)* = 4.232 in g) *N* = 8 sessions (2 brain regions, 2 mice per brain region, 2 sessions per mouse). ACA = anterior cingulate area; SS = somatosensory areas; FWHM = full width at half maximum.

### 3.6 Small-worldness increased under Dex within the ACA region only

Graph theoretical methods are highly effective in analyzing neuronal networks because each neuron can be treated as a node, with the communication between them measured to assign edges based on their degree of connectivity. In particular, small-worldness measurements are widely used to assess the structure and function of networks in biological studies (Dai et al., 2016; Zhang et al., 2021).

A heatmap depicting the Pearson’s correlation coefficient (PCC) between calcium fluorescence signal time series from 10 representative neurons in a single mouse is shown (Figures 4a,d). Examining PCC across all mice and all neurons, we found no significant change of PCC between Awake and Dex groups in either brain region (Figures 4b,e). Subsequently, we calculated the small-worldness based on these PCC values. Small-worldness was calculated from cluster coefficient and average path length. When analyzed separately, neither the clustering coefficient nor the average path length changed significantly between the Awake and Dex conditions in either the ACA or SS region (Supplementary Figure S5). In contrast, the composite small-worldness metric increased significantly in the ACA under Dex. Our findings revealed a significant increase of small-worldness within the ACA region under Dex (*p* = 0.0399, *t (3)* = 3.49) (Figure 4c). In contrast, no significant change of small-worldness was observed within SS region (Figure 4f). However, despite this difference in network structure, significant changes in calcium transients were similar for these two brain regions (Figures 3c–h). Our results show that, compared to the SS brain region, the neural network in the ACA brain region was altered, likely to maintain neuronal activity under Dex. This enhancement of the small-worldness in the ACA brain region under anesthesia indicates a reconstruction of the neural network, making the neural network more economically efficient (Bassett and Bullmore, 2009).

**Figure 4:**
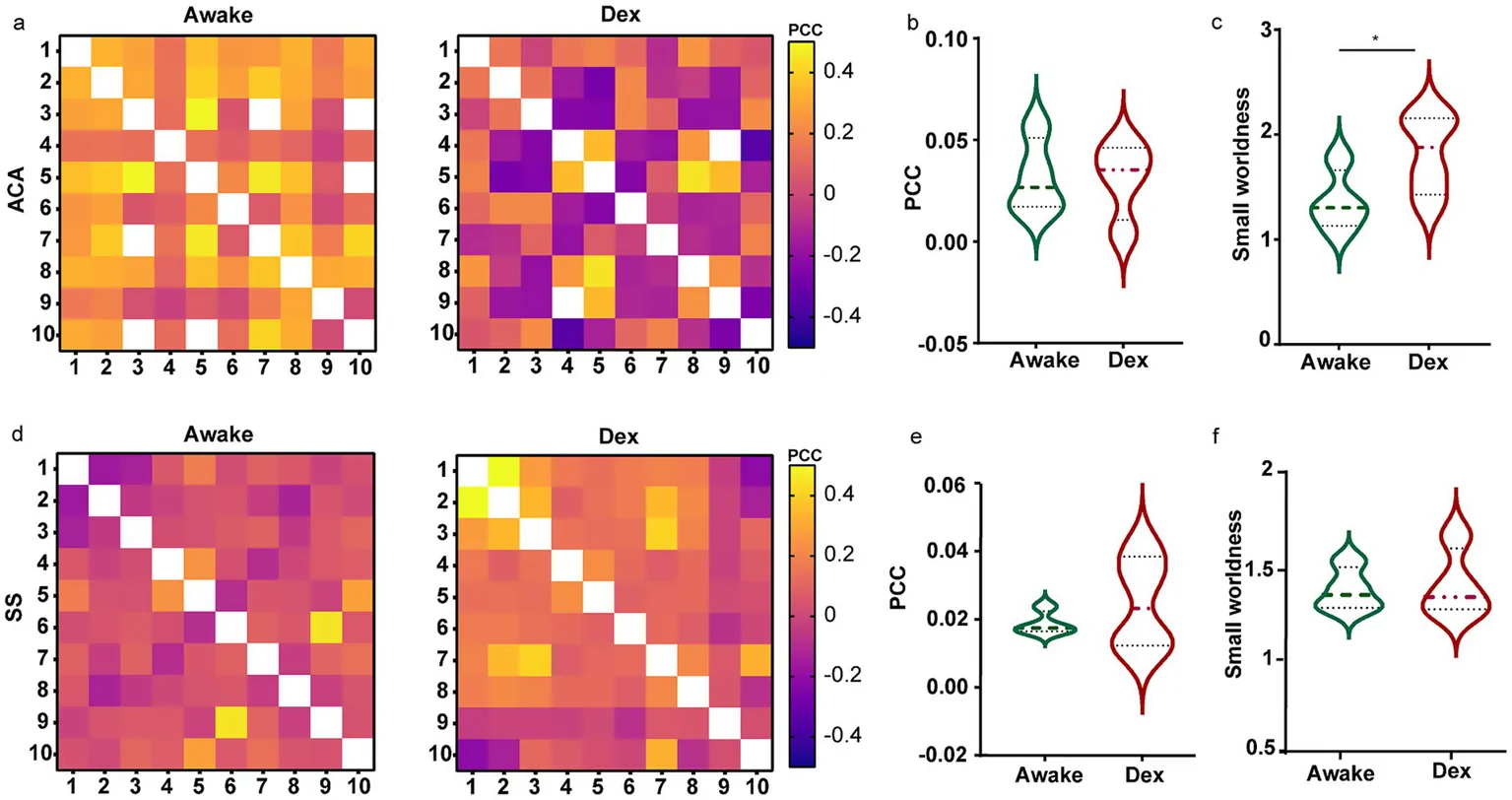
Pearson correlation coefficients of calcium signals in time series of 10 neurons and changes of small-world networks between awake and anesthesia (Dex) states. **(a,d)** Schematic diagram of the correlation coefficients between the calcium signals of 10 neurons (chosen here for illustration purposes only, actual numbers may differ) in the same mouse within the ACA brain region under awake and anesthetized states. Each row and its respective column are one representative neuron, the white squares indicating a value of 1.0. All neurons for a given region are from the same mouse. In addition, the data in the first row are all derived from the ACA brain region, and those in the second row are all derived from the SS brain region. (**b,e)** PCC under Awake and Dex conditions from ACA and SS brain regions. The upper and lower dotted lines represent the third and first quartiles, respectively, and the thick dotted line in the middle is the median. There was no significant difference in PCC under Awake and Dex conditions in either brain region. **(c,f)** Small-worldness under Awake and Dex conditions from ACA and SS brain regions, marked as in b, e Small-worldness was significantly higher in Dex group compared with the Awake group in the ACA brain region only (paired-*t* test, *p* = 0.0399, *t (3)* = 3.485). In the SS brain region, there was no significant difference between Awake and Dex groups, or even a clear trend. *N* = 24 ROI (2 brain regions, 2 mice per brain region, 2 sessions per mouse, 3 ROI per session), 8–20 neurons per ROI. PCC = Pearson’s correlation coefficient; ACA = anterior cingulate area; SS = somatosensory areas; Dex = anesthetized by dexmedetomidine.

### 3.7 Correspondence between rPWR/rCST and the underlying neural networks

Figure 3 demonstrates that within the ACA, where rPWR and rCST significantly changed, calcium transients were similar to the SS, a region where rPWR and rCST did not significantly change. This suggests that despite reduced neuronal activity, the ACA network adapts to maintain essential functions. We observed that lower rCST levels correlated with neural network efficiency (Figure 4), allowing functional integrity under energy constraints. Correspondingly, the ACA brain region is closely related to a series of critical high-energy functions, including but not limited to reward processing, decision-making, and emotional responses (Rolls, 2023).

Our findings establish a correspondence between rPWR/rCST, which characterize the match/mismatch between energy supply and neuronal activity, and properties of the underlying neural networks. The increase in macro-level energy utilization efficiency reflected by rCST is corroborated by the enhancement of small-world properties in the micro-level neural network (Table 3).

**Table 3:** Macroscopic scale of energy efficiency correspondence to neuron-level brain network reconstruction.

Factor compared	Macroscopic scale of energy efficiency	Neuron-level brain network
Parameter	rCST	Small-worldness
Alterations within the ACA brain region under the anesthesia condition	Significantly decreased	Significantly increased
Implication	Energy utilization efficiency improved	Neural network reconstruction to a more economic arrangement

ACA, anterior cingulate area; rCST, relative cost.

### 3.8 Sensitivity analysis

The sensitivity analyses showed that the rPWR and rCST results were highly stable across the tested parameter settings. For the lFCD sensitivity analysis, correlations between the alternative and primary Awake-Dex difference maps ranged from 0.990 to 1.000 for ΔlFCD, from 0.995 to 1.000 for ΔrPWR, and from 0.963 to 0.999 for ΔrCST. The directions of the regional changes were preserved across all PCC thresholds and neighborhood sizes. In ACA, ΔrPWR remained positive and ΔrCST remained negative, with unadjusted *p* values below 0.005. In SS, ΔrPWR and ΔrCST remained positive; the unadjusted *p* values for ΔrPWR ranged from 0.204 to 0.393 and those for ΔrCST ranged from 0.0486 to 0.0627. None of the SS ΔrCST results survived SGoF correction for multiple comparisons (Supplementary Table S3).

For the CMR_O_₂ sensitivity analysis, correlations with the primary difference maps were at least 0.9989 for ΔCMR_O_₂, 0.9992 for ΔrPWR, and 0.9992 for ΔrCST across the 26 alternative settings. These high correlations demonstrate that the spatial patterns of the CMR_O2_, rPWR, and rCST changes were highly consistent across the tested parameter combinations. The regional response patterns were also preserved: in ACA, the *p* values for both rPWR and rCST remained below 0.005, whereas in SS, the *p* values ranged from 0.181 to 0.435 for rPWR and from 0.014 to 0.069 for rCST. For the ACA, all of the ΔrPWR and ΔrCST *p* values < 0.05 survived SGoF correction, whereas for the SS, only 9 *p* values < 0.05 for ΔrCST and no *p* values < 0.05 for ΔrPWR survived multiple comparisons correction. Considered together with the consistency of the difference maps, these regional results indicate that variations in A, β, and α did not materially alter the rPWR/rCST results or the interpretation of the primary findings (Supplementary Table S4).

## 4 Discussion

Our research has revealed an intriguing similarity in the distribution of rPWR and rCST between humans and mice. Furthermore, compared with human studies, our mouse model allowed us to examine energy-utilization rebalancing and the neuronal network organization that may underlie it. Using this, we have found preliminary evidence of a relationship between greater efficiency of energy utilization and the reconstruction of neuronal networks into a more economical form. We have also demonstrated that the brain’s economy reorganizes at the macroscopic and microscopic levels in response to anesthesia. As the macroscopic level can be imaged non-invasively in human patients through rPWR and rCST, this suggests that application of such medical imaging methods can locate the small, hard-to-detect changes which precede larger but irreversible changes in neurological disease.

Interestingly, while lFCD and CMR_O2_ measurements were highly sensitive to the state change between Awake and Dex in all brain regions, rPWR and rCST showed significant Awake vs. Dex differences only in particular brain regions. This suggests that most brain regions maintain a similar economic profile across anesthesia, even if absolute levels of energy supply and demand increase or decrease. Our neuron-level results from the ACA strongly suggest that a biological purpose exists for its energy economics being sensitive to a change in absolute metabolism, whereas other regions such as the SS are not.

Combining measurements of brain function and metabolism is very important to the study of neurodegeneration in disease. For example, metabolic and activity abnormalities in the ACA are linked to diseases like schizophrenia, where glucose metabolism decreases more than in other regions (Fujimoto et al., 2007), and the ACA has also been implicated in neuropathic headaches (Bak et al., 2021). Calibrated fMRI, a method for assessing brain function via CMR_O2_ quantification, traditionally utilizes gas challenges (administration of exogenous CO_2_ or O_2_) to elicit hypercapnia and modulate CMR_O2_ (Davis et al., 1998; Bright et al., 2019). Herein, we employed a novel, gas-challenge-free, relaxometry-based calibrated fMRI method which has previously been used to track metabolic CMR_O2_ changes under anesthesia (Xu et al., 2022) and to contrast oxygen versus glucose metabolism in the temporal lobes of healthy individuals and epilepsy patients (Zhang et al., 2022). This approach, while effective, is constrained by lengthy scanning sessions and stringent field homogeneity requirements. However, for the purposes of calculating rPWR and rCST, it allows measurement within a single imaging session on an MRI-only scanner. Concurrently, research by Castrillon et al. has explored the energetic implications of brain function by correlating glucose metabolism with connectivity, highlighting increased energy usage in fronto-parietal networks (Castrillon et al., 2023). Unraveling the relationship between metabolic energy and neuronal activity is pivotal for brain function research.

Our results suggest that rPWR and rCST are sensitive to physiological state-dependent changes in cerebral energy balance in healthy mice. These findings may be relevant to future studies of brain disorders in which energy metabolism and functional connectivity are altered, although the present study did not test disease models directly. Alzheimer’s disease, for example, is increasingly recognized as involving glucose metabolism and mitochondrial dysfunction (Zulfiqar et al., 2019), with reduced brain glucose metabolism reported particularly in anterior and posterior default mode network regions (Guan et al., 2021). Resting-state fMRI studies have also reported connectivity decline in the default mode and sensorimotor networks (Damoiseaux et al., 2012), and altered small-world network organization has been observed in Alzheimer’s disease (John et al., 2017). Similarly, schizophrenia has been associated with impaired frontal cortical metabolism and altered prefrontal network organization (Tost et al., 2012). Together, these observations suggest that disorders such as Alzheimer’s disease and schizophrenia involve coordinated alterations in metabolic processes and functional network organization, rather than isolated changes in either metabolism or connectivity alone. Because rPWR and rCST jointly index the relationship between metabolic supply and functional demand, they may provide a useful framework for future studies examining such disorders. However, disease-model and patient-cohort studies will be required to determine whether these metrics can distinguish pathological states, track disease progression, or evaluate treatment responses.

Certain constraints exist in our approach. First, the replacement of CMR_glc_ with CMR_O2_, while avoiding FDG-PET associated radiation, is a less direct measurement that requires a relatively more complex mathematical model to interpret. Second, while an fMRI signal can be extracted from white matter under good conditions, in our study we observed a weak functional signal and extreme sensitivity to noise in the white matter fMRI, particularly in the awake condition. Thus, white matter data had to be excluded. However, stronger anesthetics that maintain an awake-like functional signal could be considered in the future [e.g., our previous work examining mouse fMRI in white matter was done under urethane (Li et al., 2024)]. Third, the two-photon calcium imaging experiment was performed in a limited number of animals and in a separate cohort from the fMRI experiment. Although awake and Dex conditions were compared within the same mice with repeated imaging fields/sessions, these repeated measurements do not replace additional animal-level biological replicates, and residual effects of cranial surgery or variability in recovery duration may have influenced calcium imaging signals. Therefore, the two-photon results should be interpreted as MRI-guided, region-specific neuronal-level evidence rather than direct within-animal or time-locked validation of macroscopic rPWR/rCST changes.

Our study suggests that rPWR and rCST are sensitive to physiological state changes, exhibiting regionally heterogeneous alterations that reflect underlying neural network reconfigurations. These findings provide a basis for future studies examining the relationship between brain function and metabolism across physiological and pathological conditions.

## Data Availability

The original contributions presented in the study are included in the article/Supplementary material, further inquiries can be directed to the corresponding authors.

## Footnotes

1
http://mialab.mrn.org/software/gift/

2
https://github.com/eshoko/COMET

3
https://github.com/soltanianzadeh/STNeuroNet

4
https://imagej.net/software/imagej/
