# Supplementary material for: Macroscopic cerebral energy efficiency corresponds to neuron reorganization in awake and anesthetized mice
Source: Front Neurosci. 2026 Oct 9;20:1949911. doi: 10.3389/fnins.2026.1949911 (PMC13690678; doi:10.3389/fnins.2026.1949911)
Supplement: Supplementary file 1 [file Supplementary_file_1.docx]

**Supplemental material**

**Supplemental Fig. 1: Workflow of MRI experiment.**

**a** Surgery was conducted to install a head post in each mouse. After recovery from the head surgery, mice were trained to acclimate to MRI scanning noise, to reduce movement caused by stress. After training for 2 weeks, a 9.4T MRI equipped with a cryo-coil was used to obtain brain images. Functional connectivity density was calculated to index brain activity. A relaxometry-based calibrated fMRI method was used to calculate CMR_O2_. **b** 2D histogram of the natural logarithm of lFCD and CMR_O2_. **c** A 45-degree counterclockwise orthogonal transformation was conducted to obtain relative power (rPWR) and relative cost (rCST). Blue points indicate rPWR > 0 and rCST < 0; gray points represent rPWR < 0 and rCST < 0; yellow points signify rPWR < 0 and rCST > 0; orange points denote rPWR > 0 and rCST > 0. Brain regions where CMR_O2_ or lFCD was significantly correlated with tSNR were removed and are depicted as black on the brain maps presented here and in following figures. Each scatter plot displays data from one mouse.

**Supplemental Fig. 2: Independent components derived from the ICA analysis of BOLD-fMRI data under consideration as nuisance regressors.** In the Awake group, components 1, 2, 4, and 8 correspond to signals originating from the cerebrospinal fluid (ventricles) and white matter. Conversely, in the Dex (anesthetic) group, components 4, 7, and 8 represent signals from white matter and cerebrospinal fluid. White matter and cerebrospinal fluid components were removed.

**Supplemental Fig. 3: 2PCI experiment time line and immunofluorescence staining.** **a** Timeline of 2PCI experiment and validation of virus injection. First, head surgery was performed, and then mice were injected with the virus in the ACA or SS region. After 1.5 weeks for recovery, mice were trained to adapt to awake two-photon imaging. After 1 week, 10 minutes of awake imaging was collected. Then dexmedetomidine was injected, and Dex images were collected. **b,c** Immunofluorescence staining of 2PCI mice. **b** is ACA brain region, virus injected at X: 2.2mm, Y: -0.34 mm, Z: 1.05 mm. **c** is SS brain region virus injected at X:0.25 mm, Y: 0.98 mm, Z: 1.0 mm.

**Supplemental Fig. 4: Mean lFCD and CMR_O2_ under Awake and Dex conditions.** **a, c** Average lFCD across all mice in Awake and Dex groups in 10 coronal slices, from caudal to rostral (n = 14). **b, d** Comparison of lFCD (b) and CMR_O2_ (d) averaged for 20 brain regions (see abbreviation list in Supplemental Table 1 and Fig. 1 caption) (paired *t*-test, SGoF corrected. * indicates *p* < 0.05, ** indicates *p* < 0.01, *** indicates *p* < 0.001, **** indicates *p* < 0.0001, the specific information on t-values and p-values is provided in Supplementary Table 2)

**Supplemental Fig. 5: a, b** **Cluster coefficient (a) and average path length(b) under Awake and Dex conditions from ACA and SS brain region.** The upper and lower dotted lines represent the third and first quartiles, respectively, and the thick dotted line is the median. Neither the cluster coefficient nor the shortest path exhibited significant changes between the awake and anesthetized conditions.

**Supplemental Table 1: List of abbreviations for brain region.**

| Abbreviation | Full name |
| --- | --- |
| ACA | Anterior cingulate area |
| AI | Agranular insular area |
| AUD | Auditory areas |
| CTXsp | Cortical subplate |
| ECT | Ectorhinal area |
| FRP | Frontal pole, cerebral cortex |
| GU | Gustatory areas |
| HPF | hippocampus formation |
| HY | hypothalamus |
| ILA | Infralimbic area |
| MB | midbrain |
| MO | Somatomotor areas |
| OLF | olfactory area |
| ORB | Orbital area |
| PAL | Pallidum |
| PERI | Perirhinal area |
| PL | Prelimbic area |
| PTLp | Posterior parietal association areas |
| RSP | Retrosplenial area |
| SS | Somatosensory areas |
| STR | Striatum |
| TE | Echo time |
| TEa | Temporal association areas |
| TH | thalamus |
| VIS | Visual areas |
| VISC | Visceral area |

**Supplemental Table 2. Comparison of experimental conditions between fMRI and two-photon calcium imaging**

| Factor | fMRI experiment | Two-photon calcium imaging | Rationale |
| --- | --- | --- | --- |
| Animal background | Male C57BL/6J mice | Male C57BL/6J mice | Both experiments were performed in male mice to maintain comparable biological background. |
| Physiological contrast | Awake vs. Dex | Awake vs. Dex | Both modalities tested the same state-dependent manipulation. |
| Dex condition | Dexmedetomidine anesthesia | Dexmedetomidine anesthesia | The same anesthetic condition was used to improve physiological comparability. |
| Awake condition | Awake imaging after head-post surgery and habituation training | Awake head-fixed imaging after habituation training | Both awake measurements were performed under trained, head-fixed conditions. |
| Regional logic | rPWR/rCST identified ACA and SS as representative regions | ACA and SS were selected for calcium imaging | Two-photon imaging was MRI-guided and targeted regions with different rPWR/rCST responses. |
| Comparison design | Awake-Dex comparison | Awake-Dex comparison | Both analyses focused on state-dependent changes rather than absolute baseline equivalence. |
| Interpretation boundary | Whole-brain macroscopic MRI metrics | Region-specific neuronal-level calcium activity | The two modalities provide convergent evidence under matched states, not simultaneous or one-to-one ROI validation. |

**Supplemental Table 3. Sensitivity of lFCD and derived rPWR and rCST results to variations in PCC threshold and neighborhood size.** The primary parameter setting was a PCC threshold of 0.25 and a neighborhood size of 2.0 mm. Pearson correlation coefficients represent the spatial correlations between the Awake–Dex difference maps obtained using each parameter setting and those obtained using the primary setting. Regional p values were calculated using paired t-tests comparing the Awake and Dex conditions in the ACA and SS and are presented as unadjusted p values. None of the SS rCST results survived SGoF correction for multiple comparisons. Formal statistical interpretation was based on the SGoF-corrected primary analysis.

| FCD PCC threshold | Neighborhood size (mm) | Parameter set | r(Delta rPWR vs primary) | r(Delta rCST vs primary) | r(Delta lFCD vs primary) | ACA rPWR p | ACA rCST p | SS rPWR p | SS rCST p |
| --- | --- | --- | --- | --- | --- | --- | --- | --- | --- |
| 0.25 | 2.00 | Primary | 1.000 | 1.000 | 1.000 | 1.69E-05 | 8.50E-04 | 0.334 | 0.027 |
| 0.22 | 1.90 | Sensitivity | 0.998 | 0.986 | 0.996 | 1.86E-05 | 2.29E-03 | 0.260 | 0.053 |
| 0.22 | 2.00 | Sensitivity | 0.997 | 0.979 | 0.994 | 2.44E-05 | 3.08E-03 | 0.239 | 0.055 |
| 0.22 | 2.10 | Sensitivity | 0.995 | 0.965 | 0.990 | 3.59E-05 | 4.85E-03 | 0.204 | 0.058 |
| 0.25 | 1.90 | Sensitivity | 1.000 | 0.999 | 1.000 | 1.75E-05 | 1.38E-03 | 0.308 | 0.054 |
| 0.25 | 2.10 | Sensitivity | 1.000 | 0.998 | 1.000 | 3.74E-05 | 3.15E-03 | 0.228 | 0.063 |
| 0.28 | 1.90 | Sensitivity | 0.995 | 0.963 | 0.994 | 1.10E-05 | 6.88E-04 | 0.393 | 0.049 |
| 0.28 | 2.00 | Sensitivity | 0.997 | 0.974 | 0.995 | 1.51E-05 | 9.73E-04 | 0.351 | 0.052 |
| 0.28 | 2.10 | Sensitivity | 0.998 | 0.986 | 0.997 | 2.50E-05 | 1.67E-03 | 0.284 | 0.060 |

**Supplemental Table 4. Sensitivity of CMR_O_₂ and derived rPWR and rCST results to variations in CMR_O_₂ model parameters.** The primary parameter setting was A = 69.3257, β = 0.80, and α = 0.20. Each parameter was varied by ±10%, yielding 27 parameter combinations. Pearson correlation coefficients represent the spatial correlations between the Awake–Dex difference maps obtained using each parameter setting and those obtained using the primary setting. Regional p values were calculated using paired t-tests comparing the Awake and Dex conditions in the ACA and SS and are presented as unadjusted p values. These p values are provided descriptively to show the consistency of the regional results across parameter settings. Formal statistical inference was based on the primary analysis with SGoF correction for multiple comparisons.

| A | β | α | ParameterSet | r_Delta_CMR_O2__vs_primary | r_Delta_rPWR_vs_primary | r_Delta_rCST_vs_primary | ACA_rCST_p | ACA_rPWR_p | SS_rPWR_p | SS_rCST_p |
| --- | --- | --- | --- | --- | --- | --- | --- | --- | --- | --- |
| 62.39313 | 0.72 | 0.18 | Sensitivity | 0.9989 | 0.9993 | 0.9994 | 3.06E-03 | 3.04E-05 | 0.194 | 0.063 |
| 62.39313 | 0.72 | 0.2 | Sensitivity | 0.9991 | 0.9992 | 0.9993 | 3.10E-03 | 3.00E-05 | 0.187 | 0.066 |
| 62.39313 | 0.72 | 0.22 | Sensitivity | 0.9992 | 0.9992 | 0.9992 | 3.14E-03 | 2.97E-05 | 0.181 | 0.069 |
| 62.39313 | 0.8 | 0.18 | Sensitivity | 1.0000 | 1.0000 | 1.0000 | 3.79E-03 | 2.62E-05 | 0.304 | 0.029 |
| 62.39313 | 0.8 | 0.2 | Sensitivity | 1.0000 | 1.0000 | 1.0000 | 3.84E-03 | 2.59E-05 | 0.294 | 0.030 |
| 62.39313 | 0.8 | 0.22 | Sensitivity | 1.0000 | 1.0000 | 1.0000 | 3.90E-03 | 2.56E-05 | 0.285 | 0.032 |
| 62.39313 | 0.88 | 0.18 | Sensitivity | 0.9995 | 0.9993 | 0.9993 | 4.61E-03 | 2.33E-05 | 0.435 | 0.014 |
| 62.39313 | 0.88 | 0.2 | Sensitivity | 0.9995 | 0.9994 | 0.9994 | 4.69E-03 | 2.30E-05 | 0.423 | 0.015 |
| 62.39313 | 0.88 | 0.22 | Sensitivity | 0.9993 | 0.9995 | 0.9995 | 4.77E-03 | 2.28E-05 | 0.410 | 0.016 |
| 69.3257 | 0.72 | 0.18 | Sensitivity | 0.9989 | 0.9993 | 0.9994 | 3.06E-03 | 3.04E-05 | 0.194 | 0.063 |
| 69.3257 | 0.72 | 0.2 | Sensitivity | 0.9991 | 0.9992 | 0.9993 | 3.10E-03 | 3.00E-05 | 0.187 | 0.066 |
| 69.3257 | 0.72 | 0.22 | Sensitivity | 0.9992 | 0.9992 | 0.9992 | 3.14E-03 | 2.97E-05 | 0.181 | 0.069 |
| 69.3257 | 0.8 | 0.18 | Sensitivity | 1.0000 | 1.0000 | 1.0000 | 3.79E-03 | 2.62E-05 | 0.304 | 0.029 |
| 69.3257 | 0.8 | 0.2 | Primary | 1.0000 | 1.0000 | 1.0000 | 8.50E-04 | 1.69E-05 | 0.334 | 0.027 |
| 69.3257 | 0.8 | 0.22 | Sensitivity | 1.0000 | 1.0000 | 1.0000 | 3.90E-03 | 2.56E-05 | 0.285 | 0.032 |
| 69.3257 | 0.88 | 0.18 | Sensitivity | 0.9995 | 0.9993 | 0.9993 | 4.61E-03 | 2.33E-05 | 0.435 | 0.014 |
| 69.3257 | 0.88 | 0.2 | Sensitivity | 0.9995 | 0.9994 | 0.9994 | 4.69E-03 | 2.30E-05 | 0.423 | 0.015 |
| 69.3257 | 0.88 | 0.22 | Sensitivity | 0.9993 | 0.9995 | 0.9995 | 4.77E-03 | 2.28E-05 | 0.410 | 0.016 |
| 76.25827 | 0.72 | 0.18 | Sensitivity | 0.9989 | 0.9993 | 0.9994 | 3.06E-03 | 3.04E-05 | 0.194 | 0.063 |
| 76.25827 | 0.72 | 0.2 | Sensitivity | 0.9991 | 0.9992 | 0.9993 | 3.10E-03 | 3.00E-05 | 0.187 | 0.066 |
| 76.25827 | 0.72 | 0.22 | Sensitivity | 0.9992 | 0.9992 | 0.9992 | 3.14E-03 | 2.97E-05 | 0.181 | 0.069 |
| 76.25827 | 0.8 | 0.18 | Sensitivity | 1.0000 | 1.0000 | 1.0000 | 3.79E-03 | 2.62E-05 | 0.304 | 0.029 |
| 76.25827 | 0.8 | 0.2 | Sensitivity | 1.0000 | 1.0000 | 1.0000 | 3.84E-03 | 2.59E-05 | 0.294 | 0.030 |
| 76.25827 | 0.8 | 0.22 | Sensitivity | 1.0000 | 1.0000 | 1.0000 | 3.90E-03 | 2.56E-05 | 0.285 | 0.032 |
| 76.25827 | 0.88 | 0.18 | Sensitivity | 0.9995 | 0.9993 | 0.9993 | 4.61E-03 | 2.33E-05 | 0.435 | 0.014 |
| 76.25827 | 0.88 | 0.2 | Sensitivity | 0.9995 | 0.9994 | 0.9994 | 4.69E-03 | 2.30E-05 | 0.423 | 0.015 |
| 76.25827 | 0.88 | 0.22 | Sensitivity | 0.9993 | 0.9995 | 0.9995 | 4.77E-03 | 2.28E-05 | 0.410 | 0.016 |

**Supplemental Table 5: Brain-region specific statistical results** (A) Results of statistically significant paired t-tests of rPWR, rCST, lFCD, and CMR_O2_.(B) Supplemental Table 3: Result of the correlation between tSNR and lFCD, CMR_O2_, rPWR, and rCST from mice in Awake state (n=14), paired t-tests. * indicate brain regions with statistically significant correlation after multiple comparison correction (SGoF). (C) Result of the correlation between tSNR and lFCD, CMR_O2_, rPWR and rCST from mice in Dex state ( n=14 ), paired t-test. * indicate brain regions with statistically significant correlation after multiple comparison ( SGoF)

|  | **(A) Statistically significant paired t-tests** | |  |  |  |  |  |  |  |  |  |  |  |  |  |  |  |  |
| --- | --- | --- | --- | --- | --- | --- | --- | --- | --- | --- | --- | --- | --- | --- | --- | --- | --- | --- |
| **Brain region** | **rPWR** |  | **rCST** |  | **lFCD** |  | **CMR_O2_** |  | **(B) Correlation with tSNR , Awake state** | |  |  |  | **(C) Correlation with tSNR, Dex state** | |  |  |  |
|  | ***p value*** | ***t* vlaue** | ***p value*** | ***t* vlaue** | ***p value*** | ***t* vlaue** | ***p value*** | ***t* vlaue** | **lFCD** | **CMR_O2_** | **rPWR** | **rCST** | **Mean fMRI tSNR** | **lFCD** | **CMR_O2_** | **rPWR** | **rCST** | **Mean fMRI tSNR** |
| **FRP** | **2.87x10^-5^** | **-5.42** | **1.9x10^-8^** | **-12.1** |  |  | **6.39x10^-6^** | **7.26** | 0.16 | -0.31 | -0.02 | -0.21 | 4.05 | 0.15 | 0.53 | 0.32 | 0.05 | 4.09 |
| **MO** |  |  |  |  |  |  | **5.22x10^-6^** | **7.4** | 0.14 | 0.06 | -0.08 | -0.25 | 3.32 | -0.2 | -0.43 | 0.41 | -0.63 | 3.07 |
| **SS** |  |  |  |  |  |  |  |  | 0.14 | 0.2 | 0.4 | -0.18 | 3.06 | -0.14 | -0.24 | -0.02 | 0.14 | 2.83 |
| **GU** |  |  |  |  |  |  |  |  | -0.52 | -0.80* | -0.72 | -0.61 | 2.88 | 0.13 | -0.1 | 0.07 | -0.03 | 2.9 |
| **VISC** |  |  | **2.25x10^-6^** | **8** |  |  | **2.62x10^-4^** | **4.96** | -0.2 | -0.52 | -0.54 | -0.3 | 2.5 | 0.24 | -0.09 | -0.16 | 0.01 | 2.64 |
| **AUD** |  |  |  |  |  |  | **2.61x10^-6^** | **7.4** | 0.07 | -0.32 | -0.13 | -0.26 | 3.14 | -0.17 | -0.12 | -0.16 | -0.24 | 3.27 |
| **VIS** |  |  |  |  |  |  | **2.38x10^-5^** | **6.39** | 0.16 | -0.33 | -0.42 | -0.11 | 1.99 | -0.17 | -0.08 | -0.09 | -0.28 | 2.09 |
| **ACA** | **1.69x10^-5^** | **-6.61** | **8.57x10^-4^** | **4.3** |  |  | **2.23x10^-4^** | **5.05** | 0.5 | 0.12 | 0.13 | -0.02 | 7.46 | -0.28 | -0.08 | 0.06 | 0.18 | 7.36 |
| **PL** |  |  | **5.81x10^-6^** | **-7.32** |  |  |  |  | 0.39 | -0.49 | -0.19 | -0.3 | 8.1 | -0.49 | 0.05 | 0.59 | -0.34 | 9.47 |
| **ILA** |  |  |  |  |  |  | **6.57x10^-3^** | **3.23** | 0.06 | -0.06 | 0.1 | 0.03 | 13.14 | -0.2 | 0.09 | -0.01 | 0.14 | 12.65 |
| **ORB** |  |  | **1.79x10^-6^** | **-6.12** |  |  | **2.00x10^-3^** | **3.85** | -0.16 | -0.33 | 0.09 | -0.01 | 7.42 | -0.24 | -0.08 | 0.38 | -0.14 | 6.93 |
| **AI** |  |  |  |  |  |  | **6.13x10^-6^** | **7.29** | -0.3 | -0.43 | 0.04 | -0.02 | 2.69 | 0.15 | 0.14 | 0.12 | 0.22 | 3.02 |
| **RSP** |  |  |  |  |  |  |  |  | 0.58 | -0.37 | -0.25 | -0.5 | 2.06 | -0.04 | 0.70* | 0.4 | 0.56 | 2.08 |
| **PTLp** |  |  |  |  |  |  |  |  | 0.5 | -0.80* | -0.61 | -0.77 | 2.03 | 0.08 | 0.49 | 0.33 | 0.56 | 2.15 |
| **TEa** |  |  | **2.34x10^-4^** | **5.02** |  |  | **7.92x10^-7^** | **8.78** | -0.09 | -0.41 | -0.24 | -0.43 | 2.14 | -0.47 | -0.24 | 0.03 | -0.45 | 2.24 |
| **PERI** | **2.40 x10^-3^** | **-3.76** | **5.27x10^-3^** | **3.34** | **9.87x10^-5^** | **5.52** | **8.45x10^-6^** | **7.07** | -0.29 | -0.41 | -0.17 | -0.4 | 2.05 | -0.12 | -0.31 | 0.13 | -0.44 | 2.22 |
| **ECT** |  |  | **1.73x10^-4^** | **5.19** | **8.59x10^-3^** | **4.3** | **4.86x10^-6^** | **7.45** | -0.28 | -0.41 | -0.32 | -0.27 | 2.44 | -0.3 | -0.41 | 0.24 | -0.55 | 2.54 |
| **OLF** | **4.5 x10^-3^** | **3.42** |  |  |  |  | **2.75x10^-8^** | **11.73** | -0.18 | -0.3 | 0.13 | -0.1 | 1.6 | -0.26 | 0.56 | 0.21 | 0.49 | 1.61 |
| **HPF** | **2.93 x10^-2^** | **-2.45** |  |  |  |  | **1.17x10^-7^** | **10.38** | -0.43 | -0.38 | 0.04 | 0.18 | 1.84 | -0.57 | -0.19 | -0.39 | -0.23 | 1.91 |
| **CTXsp** |  |  |  |  |  |  |  |  | -0.45 | -0.72* | -0.47 | -0.08 | 2.02 | -0.42 | 0.36 | 0.63 | -0.26 | 2.25 |
| **STR** | **2.28 x10^-2^** | **2.56** |  |  |  |  | **3.37x10^-4^** | **4.82** | 0.09 | -0.56 | -0.04 | -0.48 | 4.46 | -0.07 | 0.04 | -0.22 | 0.16 | 4.28 |
| **PAL** | **6.91 x10^-3^** | **3.2** |  |  |  |  | **2.98x10^-4^** | **4.88** | 0.22 | -0.42 | 0.3 | -0.59 | 6.54 | -0.06 | -0.14 | -0.12 | 0.05 | 6.54 |
| **TH** |  |  |  |  |  |  |  |  | -0.17 | -0.72* | 0.22 | -0.22 | 8.68 | -0.21 | -0.46 | -0.12 | 0.13 | 8.2 |
| **HY** | **1.70 x10^-3^** | **3.94** |  |  | **0.01** | **4.14** | **1.45x10^-4^** | **5.3** | 0.24 | -0.7 | -0.64 | -0.48 | 6.18 | 0.58 | 0.19 | -0.17 | -0.11 | 4.36 |
| **MB** |  |  |  |  |  |  | **2.77x10^-4^** | **4.93** | -0.13 | -0.37 | -0.09 | 0.1 | 5.08 | 0.02 | 0.18 | -0.24 | 0.24 | 4.77 |
|  |  |  |  |  |  |  |  | mean | 0.26 | 0.18 | 0.24 | 0.19 | 2.29 | 0.19 | 0.25 | 0.22 | 0.27 | 2.13 |
|  |  |  |  |  |  |  |  | std | 0.31 | 0.28 | 0.31 | 0.25 | 2.88 | 0.26 | 0.31 | 0.26 | 0.33 | 2.79 |
|  |  |  |  |  |  |  |  | p_value | 0 | 0 | 0.07 | 0 |  | 0 | 0 | 0.03 | 0 |  |
